# Supplementary material for: Decrypting protein surfaces by combining evolution, geometry, and molecular docking
Source: Proteins. 2019 Jun 26;87(11):952–65. doi: 10.1002/prot.25757 (PMC6852240; doi:10.1002/prot.25757)
Supplement: Supplementary file 1 — Appendix S1: SUPPORTING INFORMATION [file PROT-87-952-s001.pdf]

SUPPORTING INFORMATION
Decrypting protein surfaces by combining evolution, geometry and molecular docking

short title: Protein surface multi-usage and deformability

Chloé Dequeker[1], Elodie Laine[1,*] and Alessandra Carbone[1,2,*]

[1] Sorbonne Université, CNRS, IBPS,

Laboratoire de Biologie Computationnelle et Quantitative (LCQB), 75005 Paris, France.

[2] Institut Universitaire de France (IUF), Paris, France.

[*] corresponding authors: elodie.laine@upmc.fr, alessandra.carbone@lip6.fr

Table S1: **List of proteins in the P-262 dataset.**

PDB	resolution (Å)	chain	class	#(residues)
1aox	2.10	A	R	201
		B	R	201
1apy	2.00	A	E	161
		B	E	141
1atn	2.80	A	S	371
		D	E	258
1avf	2.36	A	E	322
		P	E	21
1avo	2.80	A	ER	60
		B	ER	140
1ck4	2.20	A	R	193
		B	R	195
1d8d	2.00	A	E	323
		B	E	407
1ef1	1.90	A	O	289
		C	O	87
1efv	2.10	A	O	312
		B	O	252
1ezx	2.60	A	SI	335
		B	SI	36
		C	E	140
1f9e	2.90	A	E	153
		B	E	89
1fnt	3.20	A	C	238
		B	C	247
		F	C	233
		H	C	196
		J	C	204
		N	C	233
1gc1	2.50	C	S	181
		G	S	297
1gl4	2.00	A	O	273
		B	S	89
1h2k	2.15	A	E	332
		S	O	24
1h6v	3.00	A	E	490
		B	E	487
		C	E	482
		E	E	491
1i7x	3.00	A	O	522
		B	O	57
		C	O	521

Continued

PDB	resolution	chain	class	#(residues)
1ibc	2.73	A	E	167
		B	E	88
1iw0	1.40	A	E	207
		B	E	209
		C	E	207
1jjo	3.06	A	O	40
		C	O	244
		E	O	33
1kfu	2.50	L	E	699
		S	ER	184
1ku6	2.50	A	E	535
		B	SI	61
1ldk	3.10	A	C	358
		B	C	366
1li1	1.90	A	S	228
		B	S	227
		C	S	224
		F	S	225
1lm5	1.80	A	O	189
		B	O	193
1lya	2.50	A	E	97
		B	E	241
1m3d	2.00	A	S	223
		B	S	224
		C	S	222
		D	S	225
		E	S	224
		F	S	223
		G	S	225
		H	S	224
		L	S	222
1mhw	1.90	A	E	173
		C	E	41
1nci	2.10	A	O	102
		B	O	96
1nme	1.60	A	E	146
		B	E	92
1nt2	2.90	A	E	209
		B	O	236
1pyo	1.65	A	E	159
		B	E	98
1r4m	3.00	B	E	418
		I	O	76
1rf3	3.50	A	E	192
		B	R	24

Continued

PDB	resolution	chain	class	#(residues)
1rkc	2.70	A	O	258
		B	O	26
1rvf	4.00	2	S	255
		3	S	236
		4	S	40
1shw	2.20	A	SI	138
		B	R	181
1us7	2.30	A	O	207
		B	O	194
1vyh	3.40	A	E	218
		C	ER	310
1wsu	2.30	A	TF	124
		B	TF	122
		C	TF	102
		D	TF	121
1y8n	2.60	A	E	374
		B	E	97
1ya5	2.44	A	O	198
		T	O	89
1ydi	1.80	A	O	256
		B	O	24
1yk1	2.90	A	R	394
		E	SI	21
1yy9	2.60	C	AB	211
		D	AB	220
2bdn	2.53	H	AB	217
		L	AB	214
2bov	2.66	A	ER	174
		B	E	208
2c0l	2.30	A	R	292
		B	O	122
2c35	2.70	A	E	129
		B	E	171
2c63	2.15	A	O	233
		B	O	233
		C	O	233
		D	O	233
2c74	2.70	A	O	235
		B	O	234
2c9w	1.90	A	O	153
		C	TF	80
2d1x	1.90	A	O	60
		B	O	59
		C	O	66

Continued

PDB	resolution	chain	class	#(residues)
2djg	2.05	A	E	114
		B	E	161
		C	E	68
2dvw	2.30	A	ER	229
		B	ER	73
2e9w	3.50	A	R	468
		C	SI	132
2e9x	2.30	A	C	144
		B	C	175
		C	C	186
		D	C	197
2gd4	3.30	H	O	234
		L	O	54
2gez	2.60	B	E	133
		C	E	166
2gix	2.02	A	R	206
		B	R	201
		D	R	205
2h0d	2.50	A	C	97
		B	E	100
2i1n	1.85	A	O	101
		B	O	102
2i32	2.70	A	O	154
		E	ER	21
2iae	3.50	A	ER	583
		B	ER	376
2jjs	1.85	A	R	116
		C	I	115
2jz3	NaN	B	TF	118
		C	TF	96
2nl9	1.55	A	O	140
		B	O	23
2nna	2.10	A	I	182
		B	I	182
2nnw	2.70	A	C	350
		B	E	227
2nvu	2.80	B	E	789
		C	E	176
2o8a	2.61	A	E	295
		I	SI	59
2odb	2.40	A	E	177
		B	E	35
2ot3	2.10	A	G	253
		B	E	157

Continued

PDB	resolution	chain	class	#(residues)
2p1m	1.80	A	O	90
		B	R	567
2pav	1.80	A	S	361
		P	S	139
2pjy	3.00	A	O	112
		B	R	108
		C	R	79
2q7n	4.00	A	R	480
		B	SI	180
2r9p	1.40	A	E	224
		E	SI	58
2rgn	3.50	A	G	324
		B	G	327
		C	G	177
2rhk	1.95	A	O	119
		C	SI	63
2uzi	2.00	H	AB	114
		L	AB	104
2v17	1.65	H	AB	222
		L	AB	214
2v8q	2.10	A	E	102
		B	E	73
		E	E	304
2vgl	2.59	A	C	600
		B	C	579
		M	C	396
		S	C	142
2vp7	1.65	A	O	66
		B	O	33
2z3q	1.85	B	R	81
		C	SI	117
2z5h	2.89	B	O	51
		I	O	39
		T	O	34
2zch	2.83	H	AB	229
		L	AB	215
		P	I	237
2zl1	2.00	A	O	119
		B	O	116
3bc1	1.80	B	O	52
		E	G	175
3bes	2.20	L	SI	133
		R	O	250
3bpl	2.93	B	R	202
		C	R	194

Continued

PDB	resolution	chain	class	#(residues)
3brw	3.40	B	G	337
		D	G	167
3bs5	2.00	A	O	83
		B	G	74
3bt2	2.50	A	E	124
		B	O	40
		H	AB	212
		L	AB	211
		U	R	259
3byh	12.00	A	S	374
		B	S	231
3c08	2.15	H	AB	217
		L	AB	206
3c5j	1.80	A	I	178
		B	I	182
3c66	2.60	A	E	529
		C	O	24
3ch5	2.10	A	G	193
		B	O	37
3cwb	3.51	A	C	443
		B	C	421
		D	O	241
		E	C	196
		F	C	100
		H	C	70
		J	C	61
		P	O	379
		T	C	79
3d0g	2.80	A	E	597
		E	O	173
3d1m	1.70	A	O	148
		D	O	99
3d2u	2.21	A	E	281
		B	O	99
3d48	2.50	P	SI	165
		R	R	195
3d85	1.90	A	AB	213
		B	AB	216
		C	SI	133
		D	SI	290
3dgc	2.50	M	SI	141
		S	R	207

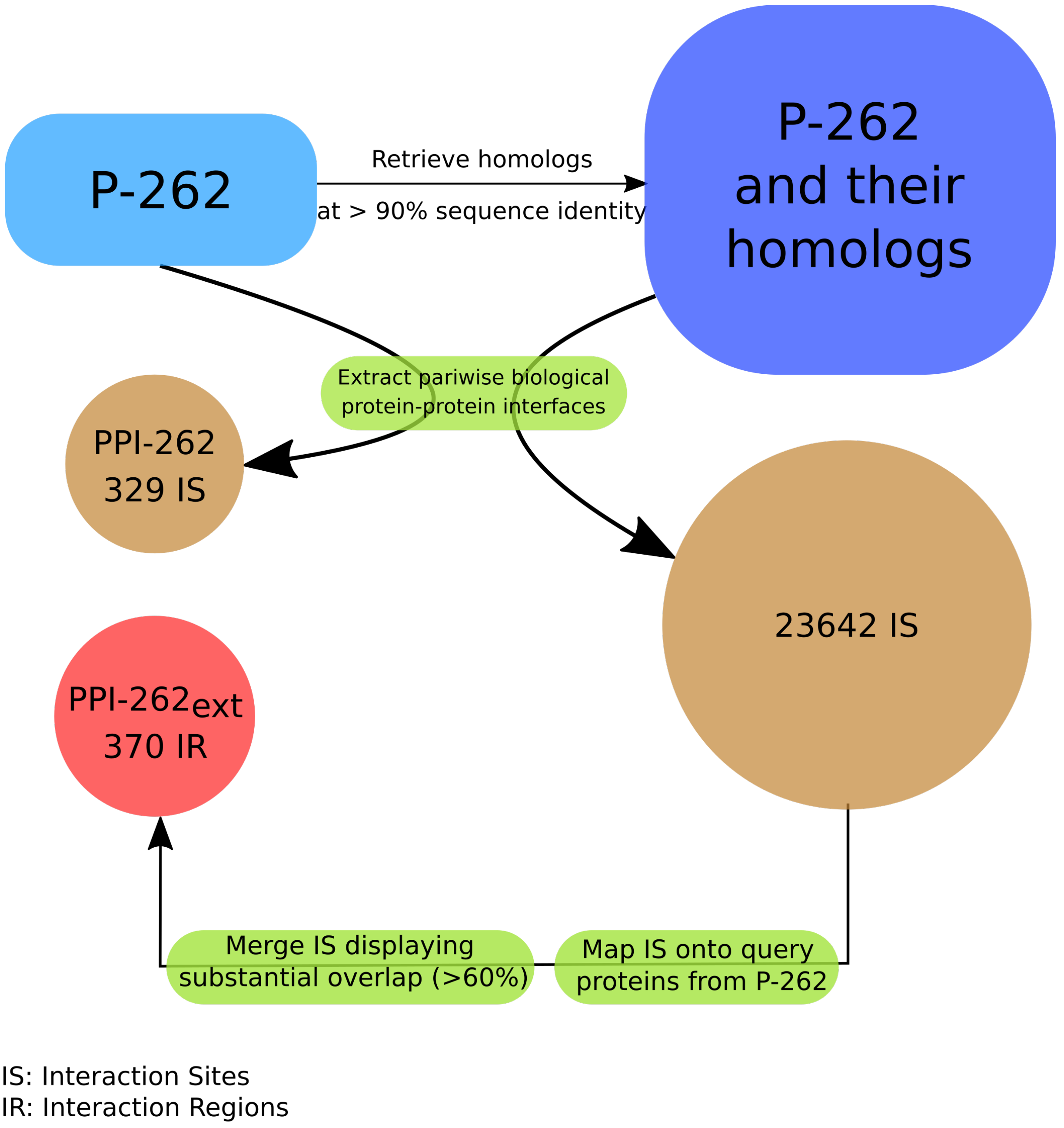

Figure S1: **Schematic representation of the protocol applied to collect interacting sites and regions.**

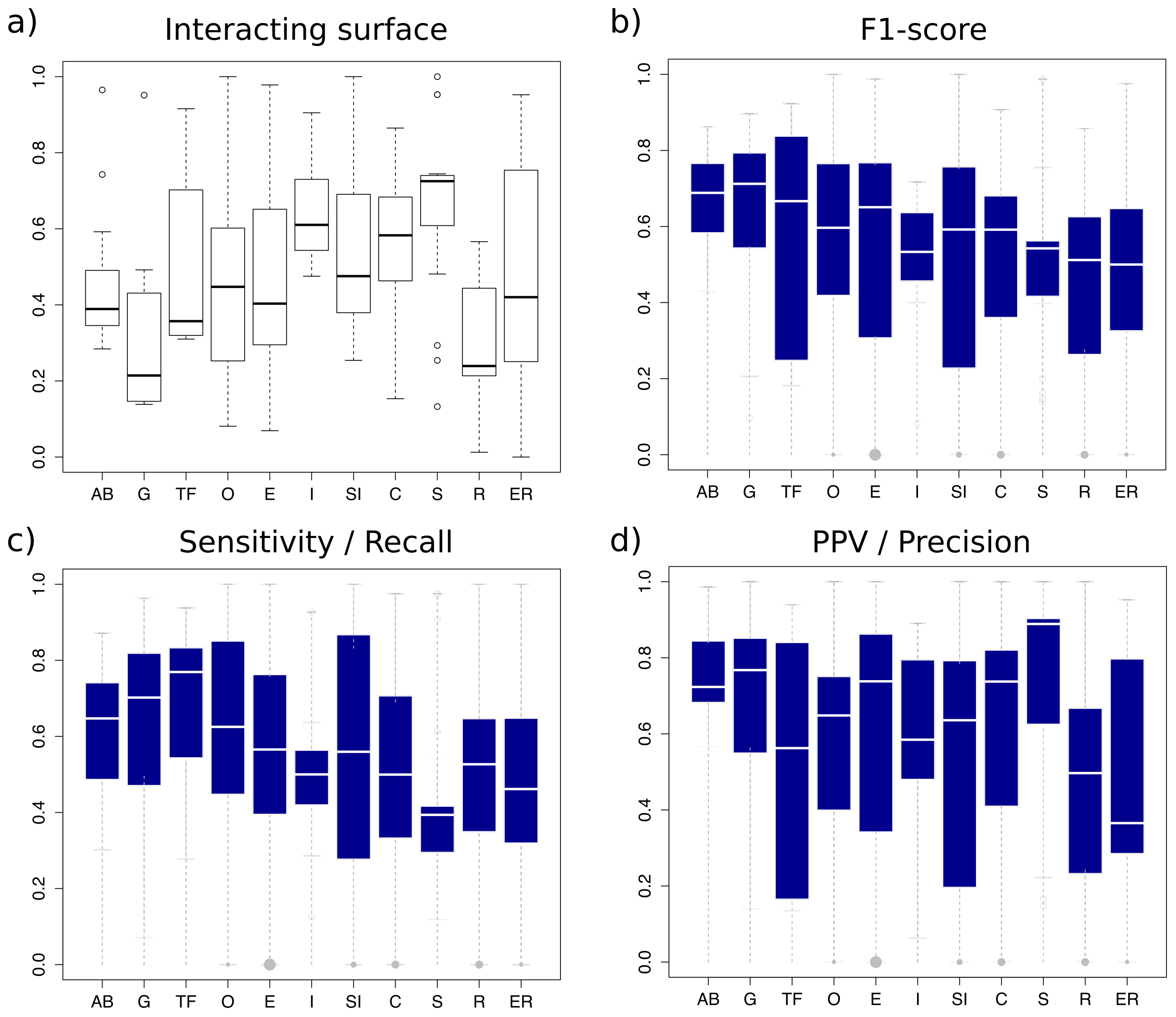

Figure S2: **Statistics computed on the different functional classes:** AB: bound antibodies (16), C: complex subunits (25), E: enzymes (60), ER: enzyme regulators (10), G: G proteins (9), I: antigens from the immune system (6), R: receptors (23), S: structural proteins (24), SI: substrates/inhibitors (16), TF: transcription factors (7) and O: proteins with other function (66). (a) Proportion of protein surface covered by experimental functional interfaces (union of IRs from $\texttt{PPI-262}_{\texttt{ext}}$). (b-d) Agreement between predicted patches and experimental IRs from $\texttt{PPI-262}_{\texttt{ext}}$. For each IR, the best-matching patch or combination of patches is retained. The performance measures are the following: (b) F1-score, (c) sensitivity (recall), (d) positive predicted value (precision). The sizes of the grey dots are proportional to the number of IRs that could not be detected at all.

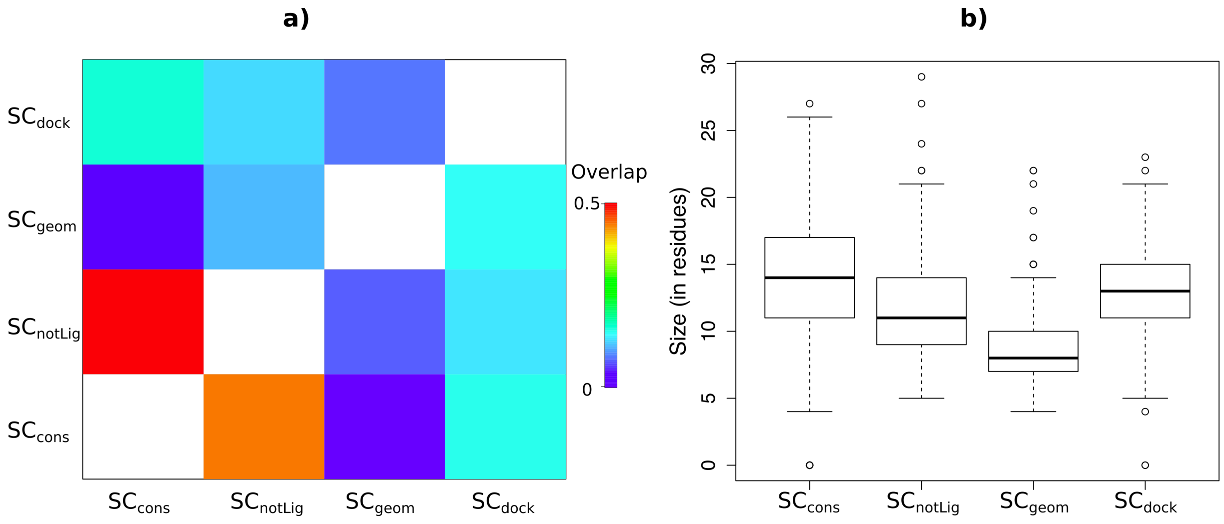

Figure S3: **Characteristic features of the seeds detected by dynJET^2.** (a) Overlap between seeds generated by the different dynJET^2 scoring schemes, averaged over all proteins from `P-262`. The overlap is computed as: $over(i,j) = \frac{|s_i \cap s_j|}{|s_i|}$, where s_i is the ensemble of seed residues predicted by the i^{th}scoring scheme. (b) Distributions of the sizes (in residues) of the seeds predicted by dynJET^2 different scoring schemes.

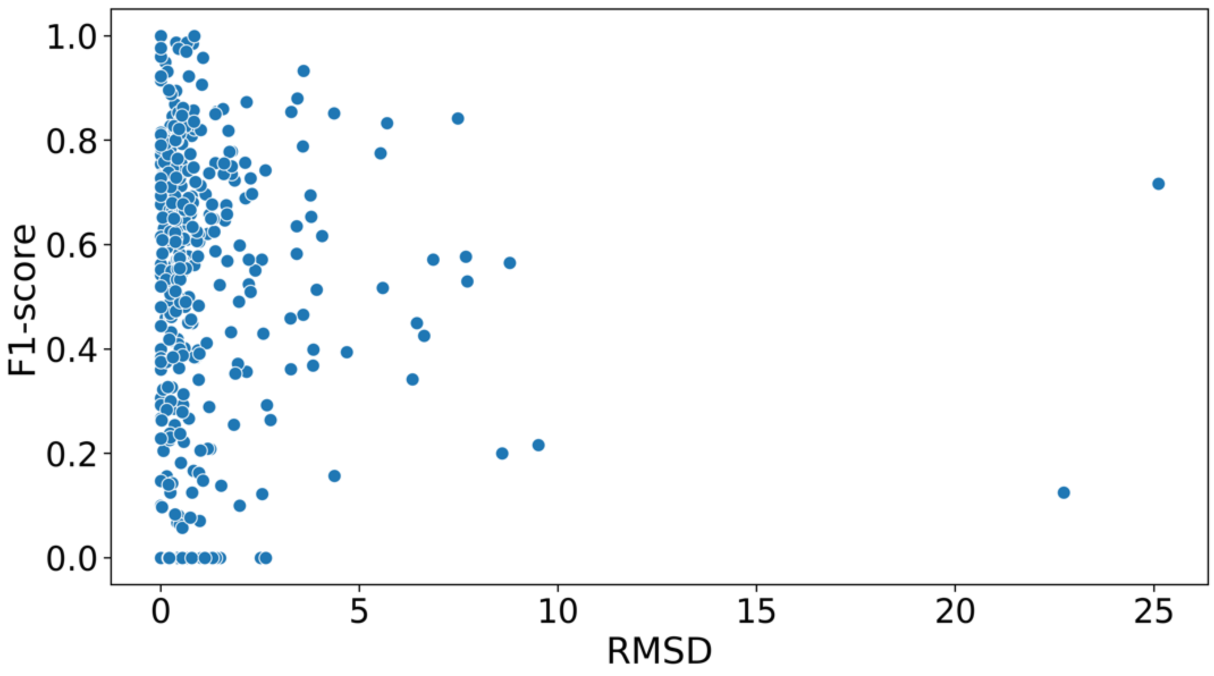

Figure S4: **Representation of the RMSD values compared to the F1-scores.** Each point represents the values obtained for one IR. The RMSD values are computed on the backbone atoms between the query structure from `P-262` and each of the homologous structures on which the IR was detected. The F1-score values correspond to the best combination of dynJET^2 predictions.
